# Simultaneous Analysis of Fenthion and Its Five Metabolites in Produce Using Ultra-High Performance Liquid Chromatography-Tandem Mass Spectrometry

**DOI:** 10.3390/molecules25081938

**Published:** 2020-04-22

**Authors:** Jonghwa Lee, Jeong-Han Kim

**Affiliations:** Department of Agricultural Biotechnology and Research Institute of Agriculture and Life Sciences, Seoul National University, Seoul 08826, Korea; jhlee006@gmail.com

**Keywords:** fenthion, fenthion oxon, fenthion oxon sulfoxide, fenthion oxon sulfone, fenthion sulfoxide, fenthion sulfone, QuEChERS, UHPLC-MS/MS

## Abstract

A simultaneous analytical method for the organophosphorus insecticide fenthion and its five metabolites (fenthion oxon, fenthion oxon sulfoxide, fenthion oxon sulfone, fenthion sulfoxide, and fenthion sulfone) was developed based on ultra-high performance liquid chromatography-tandem mass spectrometry (UHPLC-MS/MS). Five matrices (brown rice, chili pepper, orange, potato, and soybean) were selected to validate the method. The target compounds were analyzed using positive electrospray ionization in the multiple reaction monitoring mode. For the best sensitivity in regard to the detector response, water and methanol containing formic acid (0.1%) were selected as the mobile phase. The optimum extraction efficiency was obtained through a citrate-buffered QuEChERS (quick, easy, cheap, effective, rugged, and safe) method. Recovery tests were carried out at three spiking levels (*n =* 3). At all fortification levels, the accuracy and precision results were between 70% and 120% with a relative standard deviation of ≤15%. The limit of quantitation was 0.01 mg/kg, and the correlation coefficients (*r*^2^) of the matrix-matched calibration curves were >0.99. Significant signal suppression in the detector responses were observed for all matrices, suggesting that a compensation method, such as matrix-matched calibration, is required to provide accurate quantitative results. The applicability of the presented method was confirmed for the simultaneous analysis of fenthion and its metabolites in various crops.

## 1. Introduction

Pesticides are routinely used to control pests and diseases in agriculture, with the goal of high yield food production. However, residues of pesticides may remain in environments, such as food and feedstuff [1]. Regulatory authorities and governments have been working to monitor and regulate the residue level because pesticide residue in excess of the maximum residue limit (MRL) may adversely affect human health and the environment [2]. The pesticide residue that remains after application undergoes degradation or transformation into other forms by biodegradation or chemical reactions, such as hydrolysis and oxidation. The pesticide degradation products are not only detected more frequently than their parent compounds [3], but their residue amounts often exceed those of their parent compounds [4]. In addition, it has been reported that some degradation products are more toxic than their parent compounds in the case of some organophosphorus and carbamate insecticides [5].

Fenthion (*O*,*O*-dimethyl *O*-4-methylthio-*m*-tolyl phosphorothioate) is a widely used insecticide belonging to the organophosphate group. As an inhibitor of acetylcholine esterase, fenthion has been used to control a wide range of pests, such as fruit flies, mosquitoes, mites, and aphids in a variety of crops [6]. Although fenthion exhibits moderate toxicity and is classified as toxicity Class II by the U.S. Environmental Protection Agency (EPA), the adverse effect of fenthion on the environment is a controversial issue due to the high toxicity on non-target birds, aquatic invertebrates, and honey bee [7]. Moreover, it has been reported that the acute and chronic dietary risks of fenthion exceed the concern level suggested by the EPA for the general U.S population [8]. The cumulative effect of fenthion caused by its high solubility in fat tissue could be considered another threat to human health. [9]. To protect human health, animals, and the environment, continuous risk mitigation measures ought to be taken to reduce any possible risks from fenthion exposure. However, due to its broad-spectrum applicability in agriculture, fenthion is often detected in a variety of foods and environments [10,11]. Therefore, a reliable analytical method for the determination of fenthion residues is necessary to evaluate food safety and possible risks to human health.

**Figure 1:**
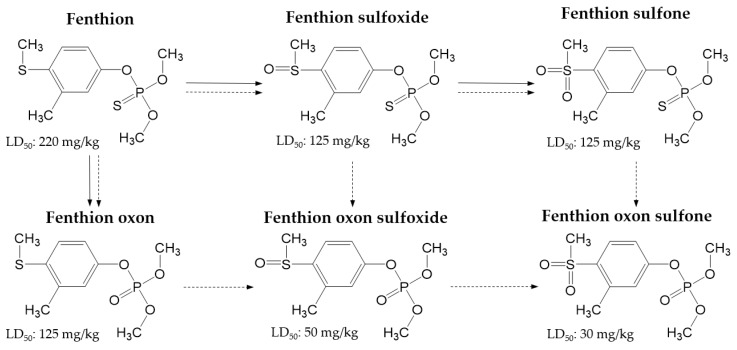
Chemical structures of fenthion and its metabolites covered in this study and their acute oral toxicities (LD_50_ for male rats) and the predicted metabolic pathway in mammals (dotted line) [14] and environmental compartments (solid line) [13].

The five degradation products, fenthion oxon, fenthion oxon sulfone, fenthion oxon sulfoxide, fenthion sulfone, and fenthion sulfoxide, are the major metabolites of fenthion, which are produced as the result of metabolism in animals, plants, and their surrounding environments [6]. The chemical structures of fenthion and the five metabolites covered in this study are shown in Figure 1, along with acute oral toxicity data (LD_50_ for male rats) [12] and the predicted metabolic pathway [13,14]. It can be seen that the toxicity of metabolites tends to increase as the metabolism or biotransformation of fenthion proceeds, through either hydrolysis or oxidation [11,15]. In particular, fenthion oxon sulfone and fenthion oxon sulfoxide (LD_50_: 50 and 30 mg/kg, respectively) are considerably more toxic than their parent compounds (LD_50_: 220 mg/kg) [12]. Despite the fact that fenthion has not been developed to be easily degraded into highly toxic oxon forms, the oxon analogs are reported to be frequently found in many environmental matrices. Previous reports have shown that fenthion sulfoxide and fenthion sulfone are the predominant metabolites found in plants [15,16]. Therefore, it is important to develop a reliable analytical method for the detection of fenthion metabolites as well as fenthion to provide more accurate data for the risk assessment of the parent pesticide. The Codex Alimentarius Commission established the MRL for fenthion as the sum of fenthion and its oxygen analogue and their sulfoxides and sulfones residues in food [17].

Since the first attempts to analyze fenthion and its metabolites in corn, grass, and milk using a gas chromatography (GC) equipped flame photometric detector (FPD) to determine their metabolic pathway, several analytical methods have been reported using a UV detector [18], nitrogen-phosphorous detector (NPD) [15,19,20,21], and FPD [22]. It was reported that analyzing fenthion sulfoxides by GC is challenging because sulfoxide can easily be oxidized and converted into a sulfone form in the injection port of a gas chromatograph. Some alternative methods, such as derivatization or oxidation of the metabolites before injecting into a gas chromatograph, have been studied to improve the sensitivity of GC [19,23]. Picó et al. [16] introduced a high-resolution mass spectrometry technique (HR-MS) to confirm fenthion metabolites in oranges using an ultra-high performance liquid chromatography-quadrupole time-of-flight mass spectrometer (UHPLC-QTOF-MS). The method was developed for the purpose of identifying and characterizing fenthion metabolites rather than performing quantitative analysis of the residues. However, in various studies carried out to date, most of the reported methods not only require complicated procedures for extraction and clean-up, which are laborious and time-consuming, but also a large volume of toxic solvents.

Due to its excellent performance and applicability, the QuEChERS (quick, easy, cheap, effective, rugged, and safe) method has become a widely used sample preparation method in pesticide residue analysis for various matrices, including crops, water, soil, and biological matrices. The QuEChERS methodology is usually an acetonitrile extraction followed by phase separation from the water layer through salting-out. Then, dispersive solid-phase extraction (dSPE), a key procedure of the QuEChERS method, is used for the removal of interferences in extracts with clean-up sorbents, such as primary and secondary amines (PSA), C_18_, and graphite carbon black (GCB). The flexibility of the QuEChERS method, which can be modified at each step, has made it more applicable to various types of matrices or analytes. Moreover, in combination with tandem mass spectrometry (e.g., GC-MS/MS and UHPLC-MS/MS), the QuEChERS method has become one of the preferred methods for pesticide residue analysis. To the best of our knowledge, no analytical method has been reported to simultaneously quantify fenthion and its five metabolites using the QuEChERS approach with UHPLC-MS/MS instrumentation.

We aimed to develop a reliable and rapid analytical method and avoid time-consuming and laborious sample preparation to provide a robust method for routine analysis of fenthion and its metabolites. The sample preparation method and instrumental conditions, including the mobile phase and parameters of multiple reaction monitoring (MRM), were carefully optimized. Validation of the developed method was performed in five matrices (brown rice, chili pepper, orange, potato, and soybean), which have their own unique characteristics, as well as having the distinction of being frequently consumed in the market. The matrix effect, which is commonly observed in instrumental analysis, was also investigated.

## 2. Results and Discussion

### 2.1. Optimization of Multiple Reaction Monitoring (MRM) Transitions 

To acquire reliable chromatographic data, optimization of the MRM transition is an essential step in tandem mass spectrometry. The process of MRM optimization is generally carried out in two steps: selecting suitable precursor ions through a full-scan analysis, followed by searching the ions derived from the selected precursor ion under various collision energies. In the case of fenthion and its metabolites, careful optimization was required since each compound has a similar structure and their molecular weights are very similar. First, the particular precursor ions from each target compound were chosen by directly injecting a standard solution into the mass spectrometer. Full-scan spectra of six compounds were obtained by direct injection of the standard solutions (Supplementary Material Figure S1). In all cases, the protonated molecular ions were readily detected as the base peak that gave the highest intensity within the scan range (50−350 *m*/*z*). In addition, ammonium adduct ions ([M + NH_4_]^+^) were found in fenthion sulfone (*m*/*z* 328) and fenthion oxon sulfone (*m*/*z* 312). Sodium adduct ions ([M + Na]^+^) were also detected in fenthion sulfoxide (*m*/*z* 317), fenthion oxon (*m*/*z* 285), and fenthion oxon sulfoxide (*m*/*z* 301). Since these adduct ions had significantly lower sensitivities compared with the base peak, the protonated molecular ions in all compounds were chosen as precursor ions. For further optimization, the product ion scan was performed over a range of collision energies from 0 to 50 eV.

The pairs of precursor and product ions with the specific collision energies that provided high sensitivity and selectivity were chosen as the final MRM transition. The optimized MRM conditions are shown in Table 1. In the process of selecting transitions, three candidate product ions were searched for from each precursor ion. It is remarkable that the same product ions were frequently found due to the similar chemical structures having the same moiety. To increase selectivity, we tried to exclude similar product ions that can cause interference between transitions. Nevertheless, it was inevitable that the same qualifying transition existed within fenthion oxon sulfone and fenthion oxon sulfoxide due to the similarity of their chemical structures. However, the retention times were significantly different and thus did not affect the quantification and qualification of each other.

**Figure 2:**
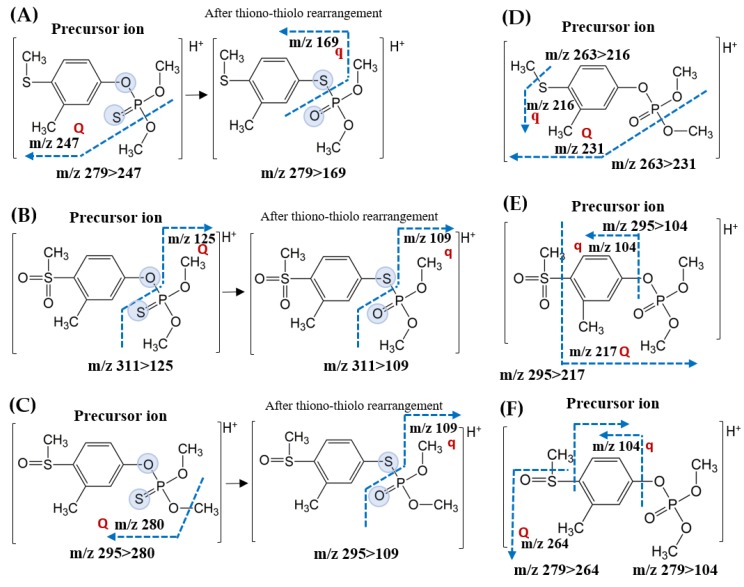
Predicted MS/MS fragmentation scheme of the precursor ions, induced by optimized collision energies: (**A**) fenthion, (**B**) fenthion sulfone, (**C**) fenthion sulfoxide, (**D**) fenthion oxon, (**E**) fenthion oxon sulfone, and (**F**) fenthion oxon sulfoxide.

No valid mass fragment ions in the mass scan spectra were observed for any compounds due to the strong intensities of abundant protonated molecular ions ([M + H]^+^) (Figure S1). Therefore, possible mass fragmentation induced by the certain collision energies were predicted. The predicted fragmentation mechanism of the precursor ions is illustrated in Figure 2. In the case of fenthion (Figure 2A), *m*/*z* 247 and *m*/*z* 169 were presumably generated by a loss of methanol and the entire phosphate group (-PO_2_CH_3_-OCH_3_), respectively. It was reported that thiono-thiolo rearrangement of the protonated molecular ion (*m*/*z* 279), which is an intramolecular rearrangement converting O-P bond to S-P bond, may occur during the fragmentation process prior to the removal of the phosphate moiety [16,24]. The precursor ions of fenthion sulfone, *m*/*z* 311, formed either *m*/*z* 125 by cleavage of P-O bond or *m*/*z* 109 by cleavage of P-S bond after the rearrangement (Figure 2B). The same mechanism forming *m*/*z* 109 was also found in fenthion sulfoxide (Figure 2C). It has been suggested that loss of a methyl radical might occur either in methoxy moiety (*O*-CH_3_ for fenthion sulfoxide) or in methyl sulfoxide moiety (SO-CH_3_ for fenthion oxon sulfoxide) [16]. Presumably, methanol was removed from the protonated precursor ion (*m*/*z* 263) of fenthion oxon to produce *m*/*z* 231, from which methyl radical was removed again from the methyl sulfide group to generate *m*/*z* 216 (Figure 2D). Interestingly, for both fenthion oxon sulfone and fenthion oxon sulfoxide (Figure 2E,F), *m*/*z* 104 ion corresponding to C_8_H_8_ was formed by the loss of dimethyl phosphate group [(CH_3_O)_2_PO_2_] with either methyl sulfonyl or methyl sulfoxide. As described previously, the C_8_H_8_ was assumed to be a cycloheptatrienyl cation, the so-called tropylium cation which can be produced by the expansion of the methylbenzene ring along with the expulsion of SO_2_ or SO during the fragmentation process [16,24,25].

### 2.2. Selection of the Mobile Phase

Various factors affect the sensitivity of the chromatographic peak in UHPLC-MS/MS. In electrospray ionization, the composition of the mobile phase is one of the important factors affecting sensitivity, along with the physicochemical properties of the target compound [26,27]. Mobile phase modifiers such as formic acid and ammonium formate are known to provide better peak resolution or retention on the chromatogram in addition to enhancing the ionization capacity [28]. In this study, three representative combinations of the mobile phase, which consisted of the pair of water and either methanol or acetonitrile with additives (formic acid and ammonium formate), were compared to increase the peak sensitivity of six compounds.

**Figure 3:**
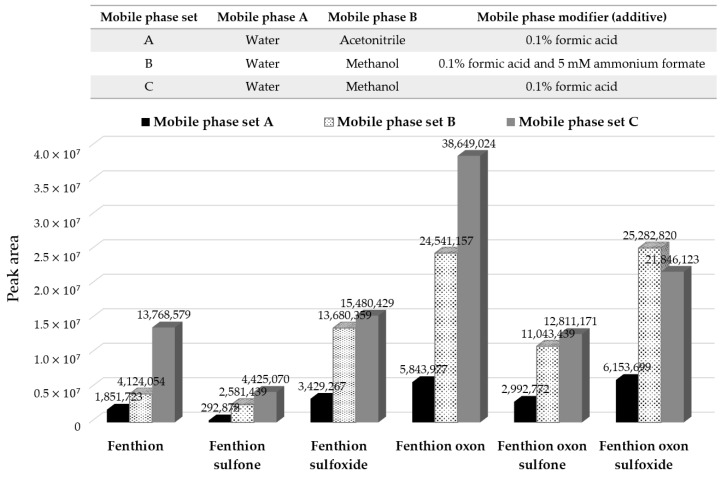
Comparison of the peak responses in standard mixtures of 0.1 µg/mL across the different mobile phases: (**A**) water and acetonitrile (0.1% formic acid); (**B**) water and methanol (5 mM ammonium formate and 0.1% formic acid); and (**C**) water and methanol (0.1% formic acid).

Figure 3 compares the signal intensities of each compound in the different mobile phases. Mobile phase set C, composed of a 0.1% formic acid solution in water and methanol, showed the greatest sensitivity for all the compounds except fenthion oxon sulfoxide (although it was still high and comparable). Interestingly, large differences between mobile phases set A and set C were observed by replacing acetonitrile with methanol in the 0.1% formic acid solution. Mobile phase set B, in which ammonium formate was added to set C, also had significantly better sensitivity than mobile phase set A but slightly lower than set B. Due to the stronger elution strength of acetonitrile, all the compounds eluted faster in mobile phase set A than in the methanol-base mobile phase sets (B and C), while there was no significant difference in the peak shape. This result indicates that the improved peak sensitivity observed in mobile phase sets B and C can be attributed to a type of organic solvent, rather than the presence of some mobile phase modifier. In addition, it was reported that the addition of ammonium formate to the mobile phase can generally increase the ion strength, resulting in an enhanced peak resolution by the decreased peak width [28]. Fenthion oxon sulfoxide had the highest signal intensity in mobile phase set B, but there was no significant difference from that in mobile phase C. As a result, mobile phase set C, which gave the best signal intensities for most of the target compounds, was selected as the mobile phase for subsequent experiments.

### 2.3. Optimization of the Sample Extraction Method

The current study focused on the development of a simultaneous analysis method that can be applied to a wide variety of matrices for fenthion and its metabolites. To find the best extraction method, a preliminary recovery test (*n* = 3) was carried out on the five matrices using representative QuEChERS extraction methods, which are frequently used for pesticide analysis. For dried commodities, such as brown rice and soybeans, hydration was essential to increase the extraction efficiency of the incurred residue along with the appropriate phase separation [29]. In addition, a 5 g sample weight was used for brown rice and soybean, which are very dry commodities, unlike chili pepper, orange, and potato, which contain a high water content (>80%). When we used 10 g of sample weight and added water, the sample volume increased by hydration, filling up the 50 mL tube. The following extraction and partitioning step did not work properly due to its bulky volume. For this reason, 5 g of sample weight were selected for brown rice and soybean, and 5 mL of water were added to adjust the water content.

**Figure 4:**
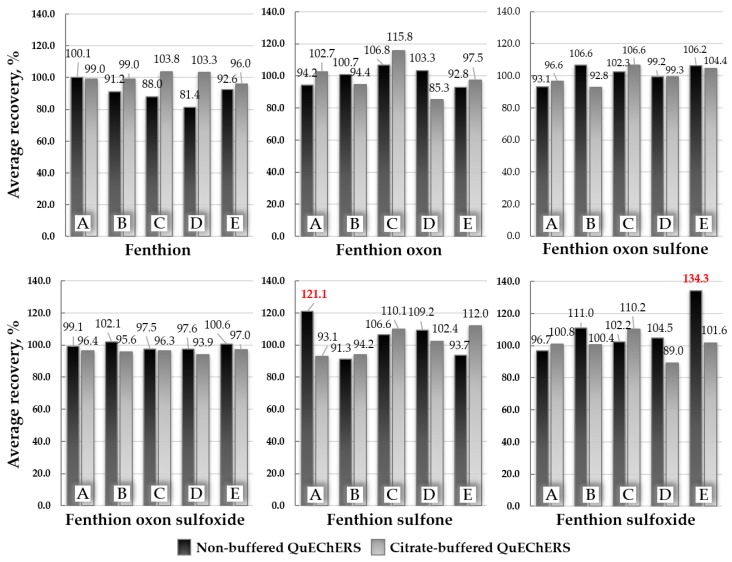
Average recoveries of fenthion and its metabolites obtained by the different extraction methods (the non-buffered QuEChERS (quick, easy, cheap, effective, rugged, and safe) method vs. the citrate-buffered QuEChERS method) at 0.1 mg/kg spiking level (*n* = 3) in various matrices: (**A**) brown rice, (**B**) chili pepper, (**C**) orange, (**D**) potato, and (**E**) soybean.

Figure 4 shows the average recovery rates of the six compounds in the different extraction methods for brown rice, pepper, orange, potato, and soybean. In all the compounds and matrices, the results from the buffered QuEChERS method satisfied the validation criteria for the recovery rate (70–120%) and relative standard deviation (RSD) (≤20%) specified in the DG SANTE guidelines [30]. Meanwhile, in the non-buffered method, some results were overestimated for fenthion sulfone (121.1%) from brown rice and fenthion sulfoxide (134.3%) from soybean, and they did not meet the validation criteria.

The citrate-buffered QuEChERS method [29] is the modified version of the original method, in which the extraction and partitioning procedures are modified by adding citrates to the extraction salts. This method can improve the stability of base-labile or acid-labile pesticides by providing a buffering condition in the extraction solvent. Since fenthion and metabolites are not pH-dependent analytes, the reasons for the unusually high recovery rates in the different matrices were not clearly elucidated. From this preliminary experiment, the citrate-buffered method that gave good recovery rates with precision for all the matrices and analytes was selected as the extraction method. The preliminary experiments already showed an acceptable extraction efficiency without any matrix interference, so the additional optimization experiment for the clean-up procedure was omitted.

### 2.4. Validation of the Analytical Method

#### 2.4.1. Specificity

**Figure 5:**
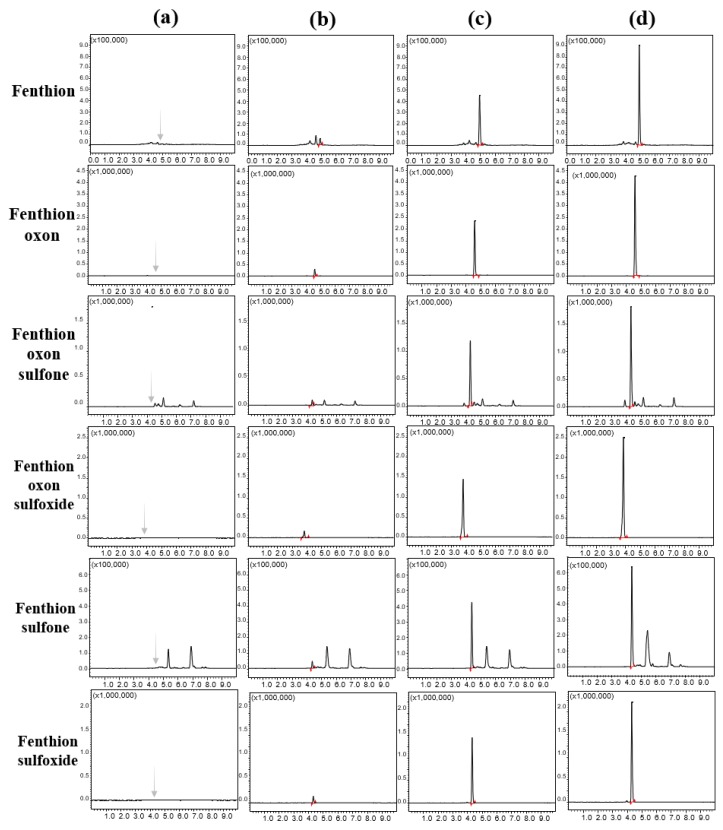
Representative UHPLC-MS/MS chromatograms for fenthion and its five metabolites: (**a**) unfortified brown rice, (**b**) brown rice fortified at 0.01 mg/kg, (**c**) brown rice fortified at 0.1 mg/kg, and (**d**) brown rice fortified at 0.2 mg/kg.

To ensure reliable data in the detector, the ion ratio between the quantifying and identifying ions obtained from the spiked sample was compared with the corresponding ion ratio obtained from the matrix-matched calibration standards. Due to the inherent co-elution of the matrix, the ratios between the different matrices did not exactly match. However, the ion ratio in the same matrix was maintained with a deviation of less than 30% as the confirmation criterion of the SANTE guidelines [30]. The system suitability of the UHPLC was also assessed by the reproducibility of the retention times. Retention time variations that can be induced by the different matrices were monitored to check the system suitability. The retention times of each compound, which were obtained from calibration curves of the five different matrices, were collected, and their deviations were investigated. As a result, the optimized method provided good system suitability, with the relative standard deviations of the retention time ranging from 0.09% (fenthion) to 1.01% (fenthion sulfone) (Supplementary Merial Table S1). Representative chromatograms for brown rice are shown in Figure 5.

#### 2.4.2. Linearity

The linearity of the matrix-matched calibration curves was evaluated by linear regression at six calibration points (0.0025, 0.005, 0.01, 0.025, 0.05, and 0.1 µg/mL). The signal intensities for all the target compounds were linear in the calibration range. In all cases, good linearity was achieved with correlation coefficients (*r*^2^) higher than 0.99, as shown in Table S2.

#### 2.4.3. Accuracy and Precision

To evaluate accuracy and precision, recovery tests were carried out on the five matrices by fortifying the six compounds at spiked levels of 0.01, 0.1, and 0.2 mg/kg. The representative commodities that had different properties were selected to demonstrate the applicability of the optimized method. Brown rice and soybean are representative dried commodities with a low water content and are included in the list of MRL for fenthion in the Republic of Korea [31]. Potato was selected because it is a frequently consumed foodstuff and because it is listed in the MRL for fenthion. Chili pepper and orange are representative commodities with a high water content and with chlorophyll-rich and acidic properties, respectively.

The accuracy and precision data obtained from the recovery experiment are presented in Table 2. All compounds gave satisfactory recoveries between 70% and 120% with an RSD ≤20%, which are the validation criteria established by the DG SANTE guidelines. The mean percent recoveries ranged from 70.3% to 100.0% (fenthion), 71.9% to 106.1% (fenthion oxon), 89.1% to 118.2% (fenthion oxon sulfone), 79.8% to 114.0% (fenthion oxon sulfoxide), 83.2% to 116.2% (fenthion sulfone), and 95.5% to 116.0% (fenthion sulfoxide) across the five matrices and all the spiked levels. In all cases, the RSDs were below 15.1%, with many below 10%. No significant differences in recovery behavior were observed among the commodities. Although the results of fenthion and fenthion oxon were satisfied according to the validation criteria, relatively low recoveries of less than 80% were found in several matrices. The wide range of recoveries from different commodities in this study was reported previously; one study showed a fenthion recovery range of 70%–128% in parsley and 87%–127% in chamomile [32].

#### 2.4.4. Limit of Quantitation

According to the DG SANTE guidelines [30], the limit of quantitation (LOQ) was defined as the lowest concentration satisfying the validation criteria for accuracy and precision. The results at all the spiking levels showed an acceptable recovery rate and RSD. Therefore, 0.01 mg/kg LOQ was determined in this study for all compounds. The LOQ obtained from this study was lower than the lowest MRL (0.05 mg/kg) for fenthion established in the Republic of Korea. The results of this study indicate that the proposed method can be applied to various commodities for the analysis of fenthion and its metabolites.

#### 2.4.5. Matrix Effect

The presence of matrix co-extracts after sample preparation frequently affects the signal response in a detector. The variable signal responses between matrices, known as “matrix effects”, may cause errors in quantitative or qualitative data, even leading to a false-negative or false-positive result. Since complete elimination of the matrix effect is difficult in multiresidue analysis, various approaches, such as alternative calibration (matrix-matched standard or isotope-labeled internal standard, or standard addition), standard addition, or dilution of the sample extract, are being used to compensate for the matrix effect [30,33,34,35,36].

**Figure 6:**
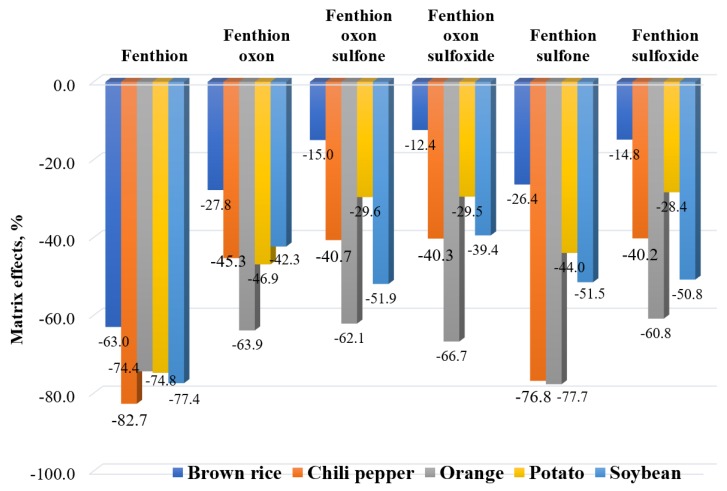
Distribution of the matrix effects in the different commodities and compounds.

In this study, matrix effects were evaluated by comparing the slope of the solvent calibration curve with the slope of the matrix-matched calibration curve obtained from each matrix. All compounds presented negative values for the matrix effect in all matrices (Figure 6), indicating that the signal responses in the matrix were suppressed compared to those from the solvent standards. Fenthion had a significant suppression, with matrix effects greater than −50%, regardless of the type of matrix. By matrix type, orange matrix showed the highest signal suppression (<−50%) in all compounds, while the lowest matrix effect was found in brown rice. This result was not surprising because signal suppression is commonly found in LC-MS/MS analysis. In general, it is known that the competitive ionization process of the target compound against the matrix components under limited proton sources cause a decreased signal response [37,38]. Given that significant signal suppression occurred from the presented method, a compensation method for matrix effects, such as matrix-matched calibration, is necessary to provide accurate quantitative results.

## 3. Materials and Methods

### 3.1. Chemicals and Reagents

HPLC grade methanol and acetonitrile were obtained from Burdick and Jackson (Muskegon, MI, USA). Mobile phase additives, formic acid, and ammonium formate (LC-MS grade, purity greater than 99%) were purchased from Sigma-Aldrich (St. Louis, MO, USA). The salt packages for non-buffered QuEChERS extraction (4 g of magnesium sulfate and 1 g of sodium chloride) and citrate-buffered QuEChERS extraction (4 g of magnesium sulfate and 1 g of sodium chloride, 1 g of trisodium citrate dehydrate, and 0.5 g of disodium hydrogen citrate sesquihydrate) were supplied by Restek (Bellefonte, PA, USA). dSPE containing 25 mg of PSA and 150 mg of sodium sulfate for clean-up were also obtained from Restek. A ceramic homogenizer to increase the homogenization efficiency was purchased from Agilent Technologies (Palo Alto, CA, USA). Certified organic produce for blank samples were obtained from local markets. High-purity (>99%) analytical standards of fenthion, fenthion oxon, fenthion oxon sulfone, fenthion sulfone, and fenthion sulfoxide were acquired from Sigma-Aldrich (St. Louis, MO, USA).

Individual stock solutions of 1.0 mg/mL were prepared by weighing a neat standard into volumetric flasks and dissolving in acetonitrile. A standard mixture solution at a concentration of 0.1 mg/mL was prepared from each stock solution, and then, the mixture working solution in a concentration range of 0.025–10 µg/mL was prepared by serial dilution using acetonitrile.

### 3.2. Instrumental Conditions

A Nexera UHPLC system (Shimadzu, Kyoto, Japan) was coupled to a tandem quadrupole mass spectrometer (LCMS-8040, Shimadzu). Electrospray ionization with MRM was operated in positive mode. The MS parameters were as follows: capillary voltage, 4.0 kV; interface temperature, 300 °C; desolvation line temperature, 250 °C; heat-block temperature, 400 °C; heating gas (air), 10 L/min; nebulizing gas (N_2_), 3.0 L/min; and drying gas (N_2_), 10 L/min. Chromatographic separation was achieved using a Waters Xbridge C18 analytical column (100 mm × 2.1 mm; 3.5 μm particle size, Milford, MA, USA). The column oven was kept at 40 °C. The mobile phase consisted of (A) deionized water and (B) methanol, both containing 0.1% formic acid. The gradient program for chromatographic separation was programmed as follows: 95% of mobile phase A for 0.5 min from the start, which was decreased to 5% (A) linearly over 2.5 min and kept for 3 min, followed by an increase to 95% (A) in 0.5 min, which was maintained for 3.5 min. The flow rate was 0.2 mL/min, and the injection volume was 5 µL. The MRM transitions and parameters were optimized carefully by the injection of individual standard solutions (0.1 µg/mL) on UHPLC without an analytical column under an isocratic flow of 1:1 (mobile phase A and B).

### 3.3. Selection of the Mobile Phases for UHPLC-MS/MS

The peak sensitivities according to the different compositions of the mobile phase were comparatively evaluated. The standard mixture solutions at a concentration of 0.1 µg/mL were injected with the following mobile phase sets: set (A), water and acetonitrile, both containing 0.1% formic acid; set (B), water and methanol, both containing 5 mM ammonium formate and 0.1% formic acid; and set (C), water and methanol, both containing 0.1% formic acid. The injections were carried out on the same analytical column and the gradient program mentioned above.

### 3.4. Optimization of the Sample Extraction Procedure

Two different QuEChERS extraction salts, namely, non-buffered QuEChERS and citrate-buffered QuEChERS extraction salts, were compared to identify the best extraction procedure. The non-buffered QuEChERS extraction salt consisted of 4 g of anhydrous magnesium sulfate and 1 g of sodium chloride; the citrate-buffered QuEChERS extraction salt consisted of 1 g of trisodium citrate dehydrate and 0.5 g of disodium hydrogen citrate sesquihydrate in addition to the non-buffered method salt. The preliminary recovery test at a spiking level of 0.1 µg/mL (*n* = 3) was conducted on the five matrices (brown rice, chili pepper, orange, potato, and soybean) using the different extraction methods. The sample extracts were cleaned-up by the dSPE and injected in UHPLC-MS/MS.

### 3.5. The Final Optimized Sample Preparation Procedure

Ten grams of the homogenized sample were weighed into a 50 mL polypropylene centrifuge tube. For rice and soybean, a 5 g sample was used, followed by the addition of 10 mL of deionized water and leaving for 30 min for hydration. Acetonitrile (10 mL) was added to the tube with a ceramic homogenizer, and the tube was shaken for 1 min at 1500 rpm using a 1600 mini G, a mechanical shaker (SPEX Sample Prep, Metuchen, NJ, USA). Then, the salt package of the citrate-buffered QuEChERS method was added and vortexed immediately for 30 s, followed by shaking in the 1600 mini G for 1 min. After centrifuging the tube for 10 min at 3000 rpm, the organic supernatant (1 mL) was transferred into a dSPE tube containing PSA (25 mg) and magnesium sulfate (150 mg). Subsequently, the dSPE tube was vortexed for 1 min and centrifuged at 13,000 rpm for 5 min. A 500 µL aliquot of the supernatant was transferred to an amber autosampler vial and mixed with 400 µL of water and 100 µL of acetonitrile. Blank matrices were also prepared using the same procedure for matrix-matched calibration.

### 3.6. Validation of the Analytical Method

The developed analytical method was validated in terms of linearity, sensitivity, accuracy, precision, and matrix effect according to the DG SANTE guidelines [30]. For validation, the recovery experiment was carried out on the five matrices at three different levels: 0.01, 0.1, and 0.2 mg/kg (*n* = 3). The accuracy was evaluated as the recovery rate (%), calculated by dividing the detected concentration by the spiked concentration. The precision was determined as the relative standard deviation (%) within three replicates. Linearity was evaluated by the correlation coefficient (*r^2^*) obtained from matrix-matched calibrations. The matrix effect was also calculated by the relative ratio between the slope of the solvent standard calibration curve and matrix-matched calibrations using the following equation:

(1) $$\mathrm{Matrix}\;\mathrm{effect},\;\%=\left(\frac{\mathrm{Slope}\;\mathrm{of}\;\mathrm{matrix}-\mathrm{matched}\;\mathrm{standard}\;\mathrm{calibration}\;\mathrm{curve}}{\mathrm{Slope}\;\mathrm{of}\;\mathrm{solvent}\;\mathrm{standard}\;\mathrm{calibration}\;\mathrm{curve}}-1\right)\times 100$$

## 4. Conclusions

The citrate-buffered QuEChERS method (EN15663) was successfully applied to analyze fenthion and its five metabolites in various commodities. Among several representative compositions of mobile phases, the combination of water and methanol containing 0.1% formic acid provided the best sensitivity for all compounds. The presented method was fully validated according to recovery tests in five representative commodities, including brown rice, chili pepper, orange, potato, and soybean, which provided acceptable accuracy and precision in all cases. The method also had good linearity and low detection limits that could determine the level below the MRLs registered in the Republic of Korea. In all the compounds and matrices, signal suppression was observed, and matrix-matched calibration was used to compensate the matrix effects to avoid errors in quantification. As a result, it was confirmed that the presented method is applicable to monitor fenthion residues, including the five degradation products, in various types of produce. However, due to the strong signal suppression observed in this study, the fact that the method requires the use of a matrix-matched calibration to compensate for the matrix effect remains a major limitation of the presented method. This drawback could be overcome by employing an isotope-labeled standard or an extra dilution approach with a more sensitive analytical instrument.

## Figures and Tables

**Table 1:** Multiple reaction monitoring (MRM) transitions and retention times for ultra-high performance liquid chromatography-tandem mass spectrometry (UHPLC-MS/MS) analysis.

Compound	t_R_^a^ (min)	Molecular Mass (g/mol)	Precursor Ion > Production (CE ^b^ Voltage)	
			Quantification Transition	Qualification Transition
Fenthion	4.92	278	279.0 > 247.0 (−13)	279.0 > 169.0 (−19)
Fenthion Sulfone	4.35	310	311.0 > 125.0 (−21)	311.0 > 109.0 (−26)
Fenthion Sulfoxide	4.30	294	295.0 > 280.0 (−19)	295.0 > 109.0 (−33)
Fenthion Oxon	4.58	262	263.0 > 231.0 (−17)	263.0 > 216.0 (−24)
Fenthion Oxon Sulfone	4.32	294	295.0 > 217.0 (−20)	295.0 > 104.0 (−28)
Fenthion Oxon Sulfoxide	3.84	278	279.0 > 264.0 (−19)	279.0 > 104.0 (−29)

^a^ t_R_: retention time; ^b^ CE: collision energy.

**Table 2:** Average recovery rates and relative standard deviations in five matrices (*n* = 3).

Compound	Spiked Level (mg/kg)	Average Recovery % (RSD ^a^%)				
		Brown Rice	Chili Pepper	Orange	Potato	Soybean
Fenthion	0.01	76.8 (5.1)	98.1 (9.9)	76.9 (5.8)	75.0 (8.1)	86.9 (0.5)
	0.1	77.1 (8.7)	82.4 (9.8)	87.1 (3.7)	95.1 (9.6)	89.5 (3.0)
	0.2	70.3 (1.0)	83.6 (5.5)	94.0 (7.4)	100.0 (9.4)	83.9 (4.5)
Fenthion Oxon	0.01	95.5 (2.8)	100.0 (2.1)	106.1 (5.2)	85.3 (4.6)	71.9 (4.4)
	0.1	96.7 (3.7)	88.7 (4.8)	97.3 (1.9)	90.2 (6.7)	94.0 (2.5)
	0.2	92.8 (5.2)	88.0 (3.9)	99.4 (5.6)	98.6 (4.7)	94.9 (11.2)
Fenthion Oxon Sulfone	0.01	102.6 (9.6)	98.1 (1.9)	102.0 (8.6)	115.9 (14.0)	89.1 (6.9)
	0.1	106.3 (9.1)	96.9 (3.4)	102.0 (5.7)	118.2 (4.4)	115.3 (0.6)
	0.2	104.5 (7.3)	93.9 (3.5)	93.8 (9.1)	105.5 (1.8)	110.3 (4.2)
Fenthion Oxon Sulfoxide	0.01	91.8 (0.8)	94.0 (1.7)	79.8 (12.9)	96.0 (2.8)	108.9 (6.0)
	0.1	92.6 (2.0)	95.1 (5.2)	94.2 (4.7)	98.0 (1.4)	114.0 (1.8)
	0.2	91.5 (2.9)	91.3 (3.1)	86.5 (9.2)	91.4 (0.2)	92.9 (3.2)
Fenthion Sulfone	0.01	101.3 (9.0)	85.8 (8.7)	83.2 (15.0)	113.9 (10.3)	90.9 (10.4)
	0.1	105.3 (7.8)	94.4 (7.7)	99.6 (8.5)	103.6 (5.9)	106.2 (2.8)
	0.2	102.8 (5.6)	88.3 (8.9)	98.4 (7.6)	116.2 (14.7)	101.7 (3.6)
Fenthion Sulfoxide	0.01	100.7 (9.9)	98.4 (1.5)	100.8 (12.1)	105.1 (8.8)	96.3 (9.7)
	0.1	104.9 (10.9)	97.1 (4.1)	102.9 (5.9)	118.1 (4.8)	116.0 (0.2)
	0.2	105.3 (7.7)	95.8 (2.8)	95.5 (8.5)	102.6 (1.2)	111.1 (4.6)

^a^ Relative standard deviation.

## Supplementary Materials

The following are available online, Table S1: The retention times of target compounds obtained from matrix matched-standards in the recovery test, Table S2: Linearity of calibration curves and limit of quantitation (LOQ), Figure S1: Full-scan spectra of target compounds: (**a**) fenthion, (**b**) fenthion sulfone, (**c**) fenthion sulfoxide, (**d**) fenthion oxon, (**e**) fenthion oxon sulfone, and (**f**) fenthion oxon sulfoxide.
