# Supplementary material for: Simultaneous Analysis of Fenthion and Its Five Metabolites in Produce Using Ultra-High Performance Liquid Chromatography-Tandem Mass Spectrometry
Source: Molecules. 2020 Apr 22;25(8):1938. doi: 10.3390/molecules25081938 (PMC7221716; doi:10.3390/molecules25081938)
Supplement: Supplementary file 1 [file molecules-25-01938-s001.pdf]

molecules

MDPI

Supplementary materials

Simultaneous Analysis of Fenthion and Its Five Metabolites in Produce Using Ultra-High Performance Liquid Chromatography-Tandem Mass Spectrometry

Jonghwa Lee † and Jeong-Han Kim *

Department of Agricultural Biotechnology and Research Institute of Agriculture and Life Sciences, Seoul National University, Seoul 08826, Korea; jhlee006@gmail.com

† Current address: Department of Veterinary and Animal Sciences, University of Massachusetts, Amherst, MA 01003, USA

* Correspondence: kjh2404@snu.ac.kr; Tel.: +82+8-880-4644

Academic Editor: Luca Campone

Received: 27 March 2020; Accepted: 20 April 2020; Published: date

Figure S1

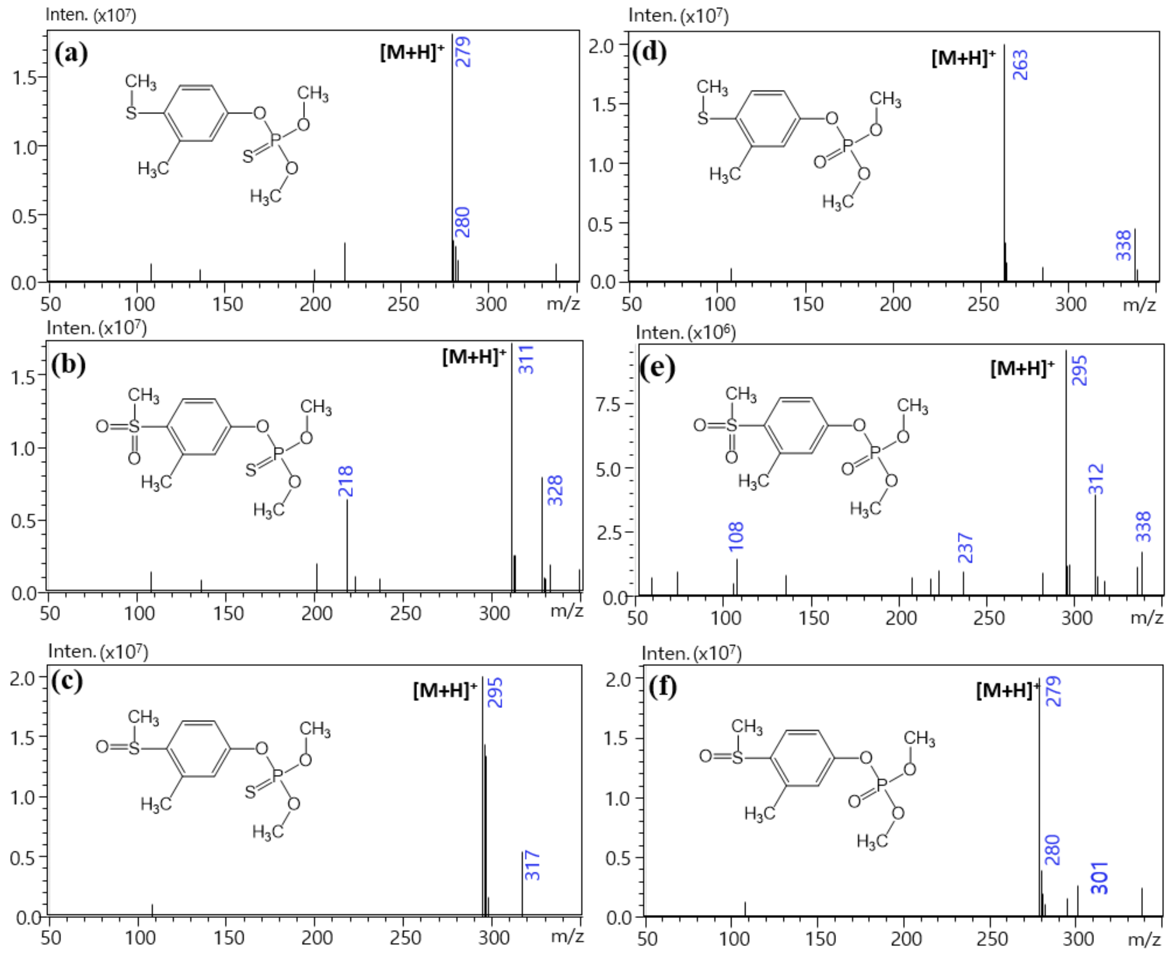

Figure 1. Full-scan spectra and their chemical structures of target compounds: (**a**) fenthion, (**b**) fenthion sulfone, (**c**) fenthion sulfoxide, (**d**) fenthion oxon, (**e**) fenthion oxon sulfone, and (**f**) fenthion.

Table S1

Table 1. The retention times of target compounds obtained from matrix matched-standards in the recovery test.

Compound	Concentration (ng/g)	Retention time (min)					RSD [a] (%)
		Brown Rice	Chili Pepper	Orange	Potato	Soybean	
Fenthion	2.5	4.931	4.924	4.928	4.933	4.926	0.09
	5	4.925	4.934	4.940	4.930	4.925	
	10	4.925	4.930	4.933	4.925	4.918	
	25	4.935	4.928	4.927	4.928	4.933	
	50	4.933	4.927	4.927	4.927	4.933	
	100	4.931	4.930	4.929	4.927	4.926	
Fenthion Oxon	2.5	4.595	4.579	4.581	4.589	4.583	0.12
	5	4.589	4.597	4.594	4.587	4.590	
	10	4.588	4.592	4.586	4.583	4.581	
	25	4.599	4.586	4.582	4.586	4.592	
	50	4.596	4.582	4.579	4.589	4.592	
	100	4.594	4.585	4.581	4.582	4.587	
Fenthion Oxon Sulfone	2.5	4.319	4.310	4.300	4.319	4.317	0.17
	5	4.313	4.327	4.315	4.315	4.322	
	10	4.312	4.322	4.307	4.311	4.313	
	25	4.324	4.315	4.302	4.314	4.323	
	50	4.321	4.311	4.299	4.319	4.323	
	100	4.320	4.314	4.301	4.312	4.317	
Fenthion Oxon Sulfoxide	2.5	3.847	3.839	3.836	3.852	3.849	0.16
	5	3.841	3.858	3.853	3.849	3.851	
	10	3.837	3.852	3.846	3.843	3.840	
	25	3.852	3.847	3.840	3.844	3.853	
	50	3.851	3.843	3.835	3.852	3.854	
	100	3.846	3.845	3.837	3.845	3.847	
Fenthion Sulfone	2.5	4.358	4.454	4.346	4.356	4.353	1.01
	5	4.351	4.472	4.362	4.353	4.358	
	10	4.350	4.467	4.354	4.347	4.350	
	25	4.362	4.460	4.349	4.352	4.358	
	50	4.360	4.456	4.345	4.358	4.360	
	100	4.358	4.458	4.349	4.349	4.355	
Fenthion Sulfoxide	2.5	4.320	4.310	4.301	4.319	4.317	0.17
	5	4.314	4.328	4.315	4.316	4.323	
	10	4.313	4.323	4.308	4.311	4.314	
	25	4.325	4.316	4.303	4.314	4.324	
	50	4.321	4.312	4.299	4.32	4.324	
	100	4.321	4.315	4.302	4.313	4.318	

[a] Relative standard deviation.

Table S2

Table S1. Linearity of calibration curves and limit of quantitation (LOQ).

Compounds	Equation and of calibration curve and regression coefficient (r^2)					LOQ/ linear range (mg/kg)
	Brown rice	Chili pepper	Orange	Potato	Soybean	
Fenthion	y = 114,175x + 96,976 (0.9988)	y = 533,14x – 22,980 (0.9966)	y = 79,029x + 200,894 (0.9977)	y = 77,822x + 323,963 (0.9901)	y = 69,631x + 100,435 (0.9999)	0.01/ 0.0025 −0.1
Fenthion Oxon	y = 429,561x + 486,651 (0.9997)	y = 325,595x – 81,123 (0.9991)	y = 215,020x + 199,220 (0.9989)	y = 316,008x + 938,595 (0.9904)	y = 343,228x + 303,493 (0.9999)	
Fenthion Oxon Sulfone	y = 168,177x + 252,294 (0.9991)	y = 117,354x + 104,961 (0.9998)	y = 74,933x + 77,456 (0.9999)	y = 139,203x + 300,416 (0.9970)	y = 95,058x + 56,183 (0.9999)	
Fenthion Oxon Sulfoxide	y = 298,367x + 567,613 (0.9990)	y = 203,361x + 193,699 (0.9995)	y = 113,375x + 95,829 (0.9998)	y = 240,089x + 411,603 (0.9989)	y = 206,187x + 225,783 (0.9994)	
Fenthion Sulfone	y = 604,12x + 47,051 (0.9999)	y = 19,017x + 34,829 (0.9988)	y = 18,309x + 29,629 (0.9999)	y = 45,473x + 89,508 (0.9930)	y = 39,812x + 35,146 (0.9998)	
Fenthion Sulfoxide	y = 206,423x + 423,117 (0.9983)	y = 144,926x + 147,003 (0.9995)	y = 94,951x + 94,495 (0.9996)	y = 173,629x + 365,365 (0.9968)	y = 119,234x + 20,211 (0.9999)	
